# Large scale meta-analysis characterizes genetic architecture for common psoriasis associated variants

**DOI:** 10.1038/ncomms15382

**Published:** 2017-05-24

**Authors:** Lam C. Tsoi, Philip E. Stuart, Chao Tian, Johann E. Gudjonsson, Sayantan Das, Matthew Zawistowski, Eva Ellinghaus, Jonathan N. Barker, Vinod Chandran, Nick Dand, Kristina Callis Duffin, Charlotta Enerbäck, Tõnu Esko, Andre Franke, Dafna D. Gladman, Per Hoffmann, Külli Kingo, Sulev Kõks, Gerald G. Krueger, Henry W. Lim, Andres Metspalu, Ulrich Mrowietz, Sören Mucha, Proton Rahman, Andre Reis, Trilokraj Tejasvi, Richard Trembath, John J. Voorhees, Stephan Weidinger, Michael Weichenthal, Xiaoquan Wen, Nicholas Eriksson, Hyun M. Kang, David A. Hinds, Rajan P. Nair, Gonçalo R. Abecasis, James T Elder

**Affiliations:** 1 Department of Dermatology, University of Michigan Medical School, Ann Arbor, Michigan 48109, USA; 2 Department of Biostatistics, Center for Statistical Genetics, University of Michigan, Ann Arbor, Michigan 48109, USA; 3 Department of Computational Medicine & Bioinformatics, University of Michigan Medical School, Ann Arbor, Michigan 48109, USA; 4 23andMe, Inc., Mountain View, California 94041, USA; 5 Institute of Clinical Molecular Biology, Christian-Albrechts-University of Kiel, Kiel 24105, Germany; 6 St John's Institute of Dermatology, Division of Genetics and Molecular Medicine, Faculty of Life Sciences and Medicine, King's College London, London SE1 9RT, UK; 7 Department of Medicine, Division of Rheumatology, University of Toronto, Toronto, Ontario, Canada M5S 1A8; 8 Centre for Prognosis Studies in the Rheumatic Diseases, Toronto Western Research Institute, University of Toronto, Toronto, Ontario, Canada M5T 2S8; 9 Department of Dermatology, University of Utah, Salt Lake City, Utah 84132, USA; 10 Department of Dermatology, Linköping University, Linköping SE-581 83, Sweden; 11 Estonian Genome Center, University of Tartu, Tartu 51010, Estonia; 12 Broad Institute of MIT and Harvard, Cambridge, Massachusetts 02142, USA; 13 Institute of Human Genetics, University of Bonn, Bonn 53127, Germany; 14 Division of Medical Genetics, Department of Biomedicine, University of Basel, Basel 4031, Switzerland; 15 Dermatology Clinic, Tartu University Hospital, Department of Dermatology and Venereology, University of Tartu, Tartu 50417, Estonia; 16 Department of Pathophysiology, Centre of Translational Medicine and Centre for Translational Genomics, University of Tartu, Tartu 50411, Estonia; 17 Department of Reproductive Biology, Estonian University of Life Sciences, Tartu 51006, Estonia; 18 Department of Dermatology, Henry Ford Hospital, Detroit, Michigan 48202, USA; 19 Department of Dermatology, University Medical Center Schleswig-Holstein, Campus Kiel, Kiel 24105, Germany; 20 Memorial University, St. John's, Newfoundland, Newfoundland and Labrador, Canada A1B 3X9; 21 Institute of Human Genetics, FAU Erlangen-Nürnberg, Erlangen 91054, Germany; 22 Ann Arbor Veterans Affairs Hospital, Ann Arbor, Michigan 48105, USA; 23 Department of Medical and Molecular Genetics, King's College London, London WC2R 2LS, UK

## Abstract

Psoriasis is a complex disease of skin with a prevalence of about 2%. We conducted the largest meta-analysis of genome-wide association studies (GWAS) for psoriasis to date, including data from eight different Caucasian cohorts, with a combined effective sample size >39,000 individuals. We identified 16 additional psoriasis susceptibility loci achieving genome-wide significance, increasing the number of identified loci to 63 for European-origin individuals. Functional analysis highlighted the roles of interferon signalling and the NFκB cascade, and we showed that the psoriasis signals are enriched in regulatory elements from different T cells (CD8^+^ T-cells and CD4^+^ T-cells including T_H_0, T_H_1 and T_H_17). The identified loci explain ∼28% of the genetic heritability and generate a discriminatory genetic risk score (AUC=0.76 in our sample) that is significantly correlated with age at onset (*p=*2 × 10^−89^). This study provides a comprehensive layout for the genetic architecture of common variants for psoriasis.

## Abstract (web summary)

Psoriasis is an immune-mediated skin disease with a complex genetic architecture. Here, Elder and colleagues identify 16 novel psoriasis susceptibility loci using GWAS meta-analysis with a combined effective sample size of over 39,000 individuals.

Psoriasis is a chronic and complex multi-genic immune-mediated skin disease^1^ affecting around 2% of European-origin individuals^2^. Previous association studies of psoriasis have identified over 60 psoriasis susceptibility loci^3^,^4^,^5^,^6^,^7^,^8^,^9^,^10^,^11^,^12^,^13^,^14^,^15^,^16^,^17^,^18^, 47 of which are associated with the risk of psoriasis in European-origin populations. These findings have greatly advanced the understanding of disease mechanisms and associated pathways. Thus, the *IL23R*, *IL12B*, *IL23A* and *TRAF3IP2* loci suggest a prominent role of the IL23 signalling pathway and promotion of T_H_17 responses; whereas the *TNFAIP3*, *NFKBIA*, *NFKBIZ*, *TNIP1* and *RELA* loci suggest dysregulation of the NFκB pathway in disease pathogenesis^19^,^20^. Approximately half (22) of the 47 European-origin loci were identified in cohorts containing a large proportion (≥50%) of samples genotyped using the Immunochip^9^,^16^,^17^,^18^, a platform that focuses on genetic variants from promising signals identified in previous association studies of autoimmune diseases^18^. However, restricting analysis to markers genotyped (∼110,000)^9^ or well-imputed (∼700,000)^17^ on the Immunochip limits exploration of the full genome for susceptibility loci. Here, we present the largest genome-wide association study (GWAS) meta-analysis for psoriasis to date in European-origin individuals. We identified 16 new disease susceptibility regions, and we also revealed various functional networks and gene regulatory signals associated with psoriasis, providing novel insights into the immunopathogenesis of psoriasis.

## Results

### Meta-analysis of GWAS studies

We gathered genotype data from both published^5^,^6^,^7^,^8^,^9^,^17^,^18^ and new cohorts, consisting of seven genome-wide association studies (GWAS) and one Immunochip data set (Supplementary Table 1). The effective sample size of the combined data set (that is, the size of a sample with equal numbers of cases and controls that possesses equivalent statistical power to the meta-analysis) is ∼40,000 samples (Supplementary Table 1); with >30,000 individuals for the GWAS component, it is about three times larger than the previous meta-analysis with the biggest GWAS component^3^,^9^,^17^. After quality control, we performed genotype phasing^21^ and imputation^22^ using haplotypes from the 1,000 Genomes project^23^. We then carried out logistic regression on each data set to determine genetic associations. Genomic inflation factors were below 1.05 for all data sets (Supplementary Table 1). Because the case definition for one of the GWAS cohorts (23andMe) was based on self-reported information, we used the risk allele frequencies for known loci in cases and controls in other cohorts to estimate the proportion of misclassified phenotypes (Supplementary Figs 1 and 2). Surprisingly, our results indicated that around 4% of the unaffected controls in the 23andMe cohort reported that they had psoriasis. To address this issue, we implemented a statistical approach^24^ to adjust the summary statistics in this dataset for bias caused by response misclassification in logistic regression^25^. As shown in Supplementary Fig. 3, this adjustment could correct for the downward bias of the estimated ORs and s.e.'s, at the cost of a substantial decrease in effective sample size.

**Figure 1:**
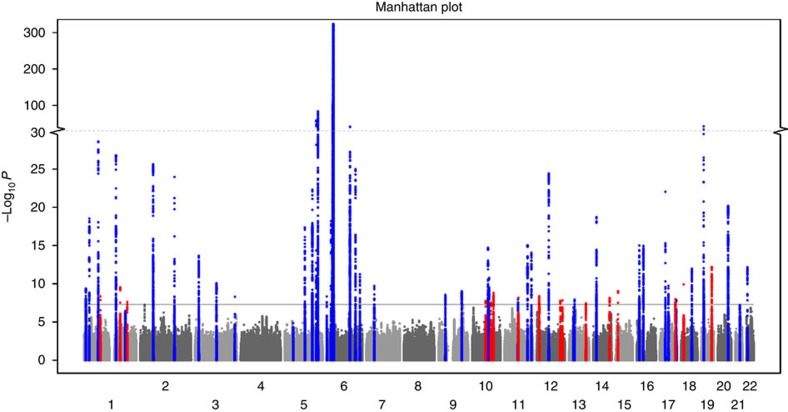
Meta-analysis results. The ‘Manhattan' plot shows the negative log *p* values of the meta-analysis. The known loci are coloured in blue; the sixteen novel loci are in red.

We performed meta-analysis of 9,113,515 markers with good imputation quality^22^ (*r*^2^≥0.7) in at least four data sets, using the inverse-variance approach^26^. Of 47 known psoriasis susceptibility loci, 42 (89%) achieved genome-wide significance (*p*≤5 × 10^-8^) (the remaining five yielded *p*≤2 × 10^-4^; Supplementary Table 2). Notably, we identified 16 new psoriasis susceptibility loci achieving genome-wide significance (Table 1 and Fig. 1; Supplementary Note; Supplementary Figs 4–6). Meta-analysis using all but the 23andMe dataset showed suggestive evidence (*p*≤2 × 10^−5^) for all new loci. Moreover, inclusion of the 23andMe data led to genome-wide significant findings for 14 of the 16 new loci, as only two loci achieved significance without the 23andMe data (Supplementary Table 3). Among the novel loci, the 19q13.33 region has been reported to reach genome-wide significance in a joint analysis of GWAS for Crohn's disease and psoriasis^15^, and in a contemporary analysis of exome array data. The adjusted ORs from the 23andMe cohort did not differ significantly from those of the other seven data sets (*p*=0.2). We also found no significant heterogeneity of ORs among studies for all new loci (Cochran's Q *p* values >0.05). Furthermore, all new loci reached genome-wide significance under a random effects model^27^.

### Genetic architecture and risk scores

**Figure 2:**
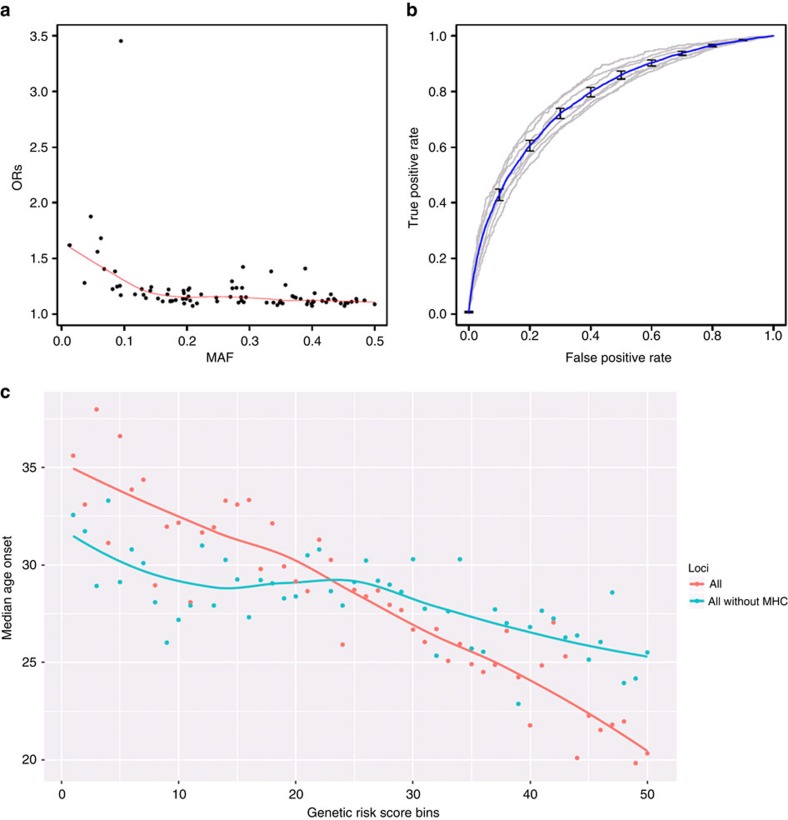
Association of psoriasis susceptibility with disease risk. (**a**) The effect size (odds ratio, OR) of the risk allele is plotted against the minor allele frequency of the signal among all susceptibility loci. (**b**) True positive rate versus false positive rate for using genetic risk score to distinguish psoriasis versus control samples. Blue line shows the averaged results among the different cohorts (grey), and the s.e. bars are also shown. (**c**) The median age-at-onset of psoriasis is plotted against different percentile bins (every 2%) of genetic risk scores for all loci (blue) or all loci without MHC (red).

By increasing the number of European-origin psoriasis susceptibility loci to 63, we were able to explore the genetic architecture of psoriasis in greater detail. Interestingly, seven (44%) of the new loci were identified using only GWAS data sets (Supplementary Table 3), as the Immunochip data does not provide good genotype coverage of these regions. Moreover, only two new loci were identified in the 186 non-contiguous regions that underwent dense genotyping in the Immunochip platform^28^. As shown for other complex traits^29^, we found that the minor allele frequencies (MAFs) of the associated signals are negatively correlated (*ρ*=−0.57; *p=*2 × 10^−8^) with the risk allele effect sizes of the disease loci (Fig. 2a; Supplementary Table 4; Supplementary Fig. 7). Thus, rs76959677 has the largest effect size (OR=1.28) and the smallest MAF (=0.04) among the new loci (Table 1). Altogether, the 63 loci account for over 28% of the estimated heritability^30^,^31^, as compared to 26% using only known loci. Our estimations are very similar to those obtained using other approaches, as shown in Supplementary Table 5. To evaluate whether the susceptibility loci could be used to discriminate between affected and unaffected individuals in our sample, we used the effect sizes and imputed dosages from our cohorts to compute genetic risk scores (GRS), and associated them with the disease status. Figure 2b shows receiver operating curves (ROC) plotting the true positive rate versus false positive rate under different GRS thresholds. The area under the curve (AUC) is 0.76, suggesting GRS has discriminative power for predicting disease status among individuals in these cohorts^5^,^6^,^7^,^8^,^9^,^15^. Age-at-onset has emerged as a key clinical and stratification feature for psoriasis^32^,^33^,^34^. To examine the correlation between age-at-onset and the GRS, we analysed 6,251 psoriatic patients for whom this information was available. Our results show that the GRS is inversely correlated with age-at-onset (Spearman ρ=−0.25; *p=*2 × 10^−89^); mean age-at-onset was 34.9 years for psoriatic patients in the lowest fifth percentile of GRS, compared to 20.4 in those in the highest fifth percentile (Fig. 2c). This correlation remains significant after removing the MHC signal from the calculation (*ρ*=−0.08; *p=*2 × 10^−11^).

### Functional interpretation of GWAS data

**Figure 3:**
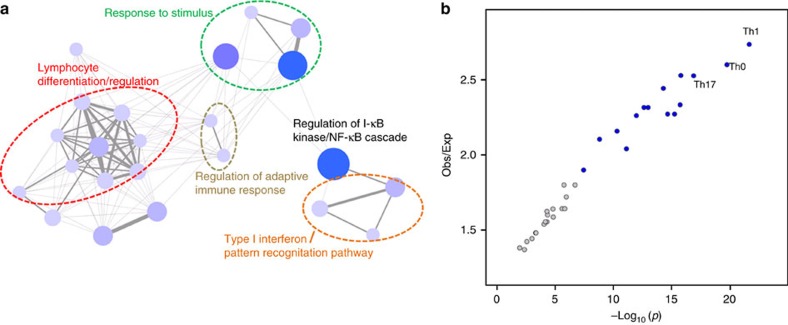
Biological inference for the psoriasis loci. (**a**) Enriched functions (nodes) among the associated loci identified using MEAGA. For illustration purposes, only functions with at least four genes overlapping with other enriched functions are shown (the full list of enriched functions/pathways is shown in Supplementary Table 6). The size of the nodes and the width of the edges correlate with the number of overlapped disease-loci and the number of shared disease-loci, respectively. Nodes with dark blue colour represents higher numbers of overlapped loci while lighter colour represents lower numbers of overlapped loci. The functional annotations for the nodes are presented in Supplementary Fig. 8. (**b**) The observed-to-expected ratio of the number of regulatory-element overlapped loci versus the enrichment *p* value. Immune cells are highlighted in blue.

To evaluate the underlying disease mechanisms responsible for these genetic signals, we applied a recently-developed algorithm termed minimum distance-based enrichment analysis for genetic association (MEAGA)^35^ to simultaneously query biological functions and pathways, as well as protein-protein interactions, for enrichment among genes mapping to the identified psoriasis loci. We found 87 significantly enriched functions/pathways (false discovery rate≤0.1, Supplementary Table 6). As expected, many of these are immune-related functions such as lymphocyte differentiation/regulation, Type I interferon, pattern recognition and response to virus/bacteria (Fig. 3a; Supplementary Fig. 8). Among the enriched functions, ‘Regulation of I-κB kinase/NF-κB cascade' (Supplementary Fig. 9) contains genes from 11 associated loci, and three of them are novel (*CHUK* in 10q24.31, *IKBKE* in 1q32.1 and *FASLG* in 1q24.3). When taken together with genes (that is, *TNIP1*, *TNFAIP3* and *NFKBIA*) mapping to known psoriasis loci that are well-appreciated as components of NF-κB signalling, our results further implicate this pathway in the pathophysiology of psoriasis. Other novel loci containing genes involved in the enriched functions identified by MEAGA include *KLRK1* (12p13.2) (‘regulation of leukocyte mediated cytotoxicity') and *PTEN* (10q23.31) (‘regulation of response to external stimulus'). We next asked whether the observed association signals are enriched among gene regulatory regions that have been mapped to different immune cell subsets in publicly-available databases^36^. Our results (Fig. 3b; Supplementary Table 7) show that the psoriasis signals are most enriched among enhancers in CD4^+^ T-helper (T_h_0, T_h_1 and T_h_17) and CD8^+^ cytotoxic T cells, in concordance with the previous study^36^. Indeed, thirteen of our novel loci (81%) either themselves harbour or are in high linkage disequilibrium (*r*^2^≥0.8) with SNPs mapping to enhancers in these cell types (Supplementary Table 8). These results complement previous studies in providing functional characterization for psoriasis-associated loci^37^,^38^. We then screened for existing drugs targeting genes from the psoriasis susceptibility loci in different drug databases^39^,^40^. We found that seven genes from six novel loci are targets for 18 different drugs (Supplementary Table 9). Interestingly, some of these drugs (that is, aminosalicylic acid^41^, mesalazine^42^ and sulfasalazine^43^) have been used to treat psoriasis in clinical practice.

## Discussion

Rather than relying on following up promising signals, here we show that the utilization of newer, less costly GWAS assays to interrogate the entire genome in follow-up samples is a cost-effective approach capable of revealing subtle genetic signals. In addition, we have implemented an approach used in epidemiology studies to adjust a misclassified binary outcome^24^. To our knowledge, this is the first large genetic association study to compare outcomes using specialist-diagnosed versus self-reported affectation and to adjust for response misclassification. Of note, we observed that disease allele frequencies and ORs were underestimated in an independent study that defined psoriasis status based on the electronic health records^44^. This may be because psoriatic lesions appear similar to other common skin diseases, including atopic eczema and seborrhoeic dermatitis, leading to misdiagnosis. Our results illustrate the importance of correcting misclassification of disease outcome as large-scale data-mining of phenotypes becomes more common. The disease-associated loci define a GRS that is capable of discriminating case-control status in our sample (AUC=0.76). Similar results have been reported in the Chinese population^45^ as well as a smaller European-origin sample^46^. In concordance with previous studies^45^,^46^, we found that the GRS is also strongly inversely correlated with age-at-onset of psoriasis, with the MHC comprising much of this effect (Fig. 2c). The strong association between HLA-Cw6 and streptococcal infection in juvenile-onset psoriasis may explain part of this association^47^. However, correlations between genetic risk allele load and age-at-onset are not universal in complex genetic disorders 48,49, and the relationship between GRS and age-at-onset needs to be explored on a disease-by-disease basis. While we did not find any disease-associated variants that alter protein structure in new loci, we demonstrated significant enrichment for genes involved in immune system function among the known and novel genetic signals. We also found significant enrichment of psoriasis genetic signals in active chromatin domains in Th1 and Th17 cells (Fig. 3). Among the individual candidates (Supplementary Note), *FASLG* encoding Fas ligand, *IKBKE* encoding IKK-ɛ, *CHUK* encoding IKK-α, *IL31* encoding the cytokine IL-31, *KLRK1* encoding NKG2D, a killer cell lectin-like receptor and *PTPN2* encoding T-cell protein tyrosine phosphatase, all play prominent roles in T-cell activation, signalling and/or effector function. By guiding further functional investigation into the roles of these variants in the regulation of their target genes, as well as further functional investigation of these targets, these results will serve as an important framework guiding future research into the pathogenesis and treatment of psoriasis.

## Methods

### Data sets

The collection of samples for the five GWAS (PsA, CASP, Kiel, Genizon and WTCCC2) and the Immunochip dataset were described previously^5^,^6^,^7^,^9^,^18^. The new Exomechip cohort, consisting of 6,463 genetically independent psoriasis cases and 6,096 unrelated controls of European Caucasian descent collected in North America and Sweden, was genotyped using the Affymetrix Axiom Biobank Plus Genotyping Array at the Affymetrix facility (Santa Clara, CA). All human subjects provided written informed consent and were enrolled according to the protocols approved by the institutional review board for human subject research of each institution, in adherence with the Declaration of Helsinki principles. The basic array contains 246,000 genome-wide markers, 265,000 exome coding SNPs and indels, and 95,000 eQTL, pharmacogenomic and novel loss-of-function variants, which was supplemented by addition of 77,000 custom markers consisting of (1) 50,000 novel rare variants identified in targeted sequencing of known psoriasis loci, (2) 15,000 additional evenly distributed markers to enhance GWAS coverage, (3) 10,000 1,000 Genome variants in ENCODE-predicted regulatory regions for normal human epidermal keratinocyte (NHK) cell lines, (4) 1,000 markers associated with other autoimmune diseases and (5) 1,000 markers representing skin eQTLs identified from our analyses of the psoriatic skin transcriptome. Psoriasis cases in these cohorts were dermatologist-diagnosed, and all studies were approved by the ethical committees of their respective institutions. In this study, we did not segregate psoriasis subphenotypes (that is, psoriatic arthritis, cutaneous-only psoriasis^18^); therefore our cohorts include psoriasis cases that might have developed psoriatic arthritis, and this is especially true for the PsA GWAS, in which all included cases have psoriatic arthritis.

### Quality control

For each dataset, we removed samples with high missingness (>2%) or a high inbreeding coefficient (|*F*|>0.03), and we also removed markers with low call rate (<95%), with more than two alleles, or that failed Hardy Weinberg equilibrium (*p*<1 × 10^-6^). We identified duplicated or highly related pairs (that is, first and second degree relatives) of individuals among our data sets using independent markers outside of the known psoriasis susceptibility loci (‘null markers')^9^; this includes samples that were genotyped in multiple cohorts (for example, the same sample might be genotyped in both the CASP GWAS or Exomechip cohorts). When related or identical pairs were identified in different data sets, we preferentially kept the sample from the genotyping platform with the higher number of markers with genome-wide coverage (Supplementary Table 1). We used the independent (that is, ld-*r*^2^<0.2) markers that are outside the known psoriasis loci to compute the principal components for each data set; and for the Immunochip data set, since the platform is enriched with markers from the immune-associated regions, we first conducted a meta-analysis using the CASP, Kiel, and WTCCC2 cohorts and identified independent markers which have meta-analysis *P* values >0.5 as ‘null markers' to compute the principal components. We then used principal components to remove the population outliers to ensure all analysed individuals were of European ancestry^9^.

### 23andMe cohort

The 23andMe cohort was drawn from the customer base of 23andMe, Inc., a personal genetics company. The samples from this cohort were genotyped on one of four platforms: the V1 and V2 platforms were variants of the Illumina HumanHap550 BeadChip with additional custom content; the V3 platform is a variant of the Illumina OmniExpress+ BeadChip, with custom content; the V4 platform is a fully custom design, including lower redundancy subsets of V2 and V3 SNPs with coverage of low allele frequency coding variants, as well as 570,000 additional SNPs. Research participants included in the cohort provided informed consent and answered surveys online according to the 23andMe human subject protocol, which was reviewed and approved by Ethical & Independent Review Services, a private institutional review board. The ‘psoriasis' phenotype combines self-reported psoriasis diagnoses from several sources available on the 23andMe website: (*i*) Medical History Survey; (*ii*) Roots into the future intake form; (*iii*) research snippet. There are three choices (yes, no, not sure) for each psoriasis-related question from each source. We merged the yes/no responses from these questions, with inconsistent responses scored as missing: cases have at least one positive response and no negative responses, and controls have at least one negative response and no positive responses. We also derived responses from two additional questions derived from the IBD Community Survey and Health Intake Form, regarding whether the individual has been diagnosed with psoriasis to define cases (when any response is a yes) and controls (when it is not a case and at least one response is control).

### Imputation and association

We performed haplotype phasing^21^ and imputation^22^ for each dataset. For imputation, we used haplotypes from all populations in the 1,000 Genomes Project phase 1 (release 3) as a reference panel^23^. We then analysed markers with imputation quality greater than 0.7 in at least half (that is, 4) of the data sets. For each data set, we performed logistic regression using top principal components and data collection center indicator variables as covariates to correct for population stratification. We computed the inflation factor (λ) using the ‘null markers' for the genomic control analysis (Supplementary Table 1).

### Proportion of true positives among 23andMe psoriasis cases

For each of the previously identified signals from the known psoriasis loci, we compared the risk allele frequencies in cases and controls estimated from our dermatologist diagnosed-based data^9^ with those estimated by the 23andMe cohort. The RAFs in cases from the dermatologist-diagnosed cohorts are systematically higher than those in the 23andMe cohort (34 out of 36 loci listed in Tsoi *et al*.^9^ manifested RAF_case_Tsoi(2012)_ higher than those estimated in RAF_case_23andMe_); while the RAFs in controls are highly concordant (Supplementary Fig. 1). We hypothesized that some of the defined cases are false positives (that is, the individuals do not actually have psoriasis). Assuming the defined cases from the 23andMe cohort contain a mixture of true cases and controls, we would get:


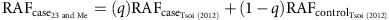


where *q* is the proportion of true positives. The proportion could then be estimated as:


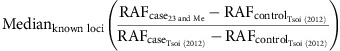


We estimated that *q=*0.36. Ignoring the misclassification of psoriasis phenotype, the 16,120 self-reported cases and 254,909 controls of the 23andMe cohort yield an estimated disease prevalence of 5.9%. But if we assume *q=*0.36 and correct for misclassification, then we would obtain a 2.1% prevalence (Supplementary Table 1), matching the disease frequencies estimated for European-origin populations^50^.

### Adjustment for misclassification of 23andMe cases

We employed Duffy's approach to adjust odds ratios and s.e.'s for bias caused by response misclassification in logistic regression^24^. If β* and *V*(β*) are the naive log OR and its variance for the misclassified case-control data, then by Duffy's method the corrected log OR and its variance can be estimated as:


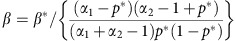


and


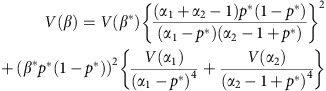


Parameters *α*_1_ and *α*_2_ are the sensitivity and specificity of the binary classification. For the 23andMe sample we assumed *α*_1_=1 (that is, all true cases were reported as such); using *q*=0.36, *α*_2_ could then be estimated as 0.9611. *V*(*α*_2_) was estimated to be 2.67 × 10^−6^ using Monte Carlo simulation based on the observed RAFs for 32,240 case chromosomes from the 23andMe cohort and for 21,176 case and 45,612 control chromosomes from our previous study^9^. *V*(*α*_1_) was assumed to be 0. The observed case prevalence in the sample (p*) is 0.0595. Because p* is small, deviations of our assumptions for *α*_1_ and *V*(*α*_1_) from their true values have little impact on the resulting estimates of β and V(*β*), which was verified by sensitivity analysis for a broad range of both parameters (that is, *α*_1_=0.5−1.0 and V(*α*_1_)=0−0.001).

### Meta-analysis and effective sample size calculation

We used the inverse-variance approach implemented in METAL^26^ to perform meta-analysis across the eight data sets. The effective sample size for each dataset (except for the 23andMe study) was computed as: 
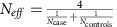
. The effective sample size approach was computed for each cohort to provide an estimate of the sample size corresponding to an equal case/control balance, and has the same statistical power to identify true association. To compute the effective sample size for the 23andMe study, we first determined the asymptotic relative efficiency (ARE) of the Duffy-corrected log OR to the log OR that would have been obtained if there had been no misclassification of disease phenotype. Because both of these OR estimators are convergent and asymptotically unbiased, the ARE for these two parameters (and their corresponding Wald chi-square test statistics) is equal to the ratio of their variances. We determined this variance ratio by simulation. We bootstrap sampled one of our largest studies with dermatologist-diagnosed phenotypes (the PAGE Immunochip study) to create 25 data sets mimicking the 23andMe study; that is, each simulated dataset had 5,803 true cases, 10,317 false cases, and 254,909 true controls. For each of 28 independent known psoriasis loci with adequate significance of association in the PAGE Immunochip (p<0.001), we determined the mean variance across the 25 data sets for the Duffy-corrected ORs from analysis of the misclassified responses and also the mean variance for the ORs from analysis of the true responses. The ARE for each locus was the ratio of these two mean variances, and the median ARE for the 28 loci was 0.35. The effective sample size for the 23andMe study after using Duffy's adjusted approach was then estimated by multiplying the effective sample size under no misclassification (22,715=4/(1/5,803+1/265,226) ) by the ARE, yielding an estimate of 7,950 (Supplementary Table 1).

### Analysis of psoriasis loci

We performed comparisons between the estimated risk allele ORs versus MAFs of the best associated markers for each of the 63 loci. In a recent fine-mapping meta-analysis study (manuscript in preparation), we observed that a substantial proportion of psoriasis loci harbor secondary independent signals, and due to the linkage disequilibrium structure the effect sizes from the primary signals could be over/under estimated if the risk allele of secondary signals tend to be on the same haplotype of the risk/non-risk alleles of the primary signals, respectively. Therefore, for each published locus with multiple signals, we computed the ORs by conditioning on the other independent signal(s) (that is, as covariates) from the same locus. These values were also used in the calculation of the variance in liability explained^31^. We next computed the genetic risk score using the effect sizes and imputed dosage data for each signal. Let the OR of the risk allele in signal *i* be OR_*i*_, and the imputed dosage/genotypes for the risk allele be *d*_*i*_, the genetic risk score for an individual among all independent associated signals is computed as:


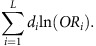


### Enrichment analysis

We analysed our GW-signficant loci using MEAGA^35^. MEAGA employs a graphical algorithm to measure the closeness between gene-set overlapping genes in the biological interactome, to identify the enriched functions/pathways among the 63 psoriasis loci. We used the protein-protein interaction data from BioGRID^51^ for generating the interactome, and the nine million markers examined in this meta-analysis as background under the default setting in MEAGA for the enrichment analysis. 50,000 samplings were used for *P* value estimation. We then sought to understand whether the psoriasis signals are enriched among regulatory elements in different cell types, as predicted by H3K27ac chromatin marks. We utilized the active enhancers identified from a recent study aiming to use epigenomics data to fine-map genetic susceptibility loci for complex autoimmune diseases^36^. The 33 cell types under study included: T_naive_, T_mem_, T_reg_, Th_stim_, Th17, Th1, Th0, Th2, CD8_naive_, CD8_mem_, Monocytes, B cell, Lymphoblastoid, B centroblast, CD34^+^, K562, Inferior temporal lobe, Angular gyrus, Mid-frontal lobe, Cingulate gyrus, Substantia nigra, Anterior caudate, Hippocampus middle, Colonic mucosa, Duodenum mucosa, Adipose, HepG2, Liver, Pancreatic islets, Kidney, Human skeletal muscle myoblasts (HSMM), NH osteoblast, Chondrogenic diff. We performed enrichment analysis by first enumerating the number of associated loci that overlap or are in linkage disequilibrium (LD) (*r*^2^≥0.8) with markers in regulatory elements, and then comparing that with the expected number of overlaps. The expected numbers were estimated by randomly sampling markers from the meta-analysis matching the LD-block length, MAF, and the number of genes in the LD-block, and counting the number of times these null markers overlap/in LD with the regulatory elements.

### Drug databases

We downloaded data from PharmGKB^40^ and Drugbank^39^, and searched for drugs with potential gene targets from these databases.

### Data availability

The data of the Exomechip cohort is available in dbGap (phs001306.v1.p1). The GWAS statistics from the 23andMe cohort can be requested by applying to the 23andMe collaboration program.

## Additional information

**How to cite this article:** Tsoi, L. C. *et al*. Large scale meta-analysis characterizes genetic architecture for common psoriasis associated variants. *Nat. Commun.* **8,** 15382 doi: 10.1038/ncomms15382 (2017).

**Publisher's note:** Springer Nature remains neutral with regard to jurisdictional claims in published maps and institutional affiliations.

## Supplementary Material

Supplementary Information Supplementary Figures, Supplementary Tables, Supplementary Notes and Supplementary References

## Figures and Tables

**Table 1:** Newly identified psoriasis associated loci.

**Chr**	**Pos**	**Marker**	**RA**	**NRA**	**RAF**_**case**_	**RAF**_**cont**_	**ORs**	**P value**	Direction*	**Nearby genes**
1	78450517	rs34517439	A	C	0.13	0.12	1.18	4.43 × 10^−9^	+?++++?+	*FUBP1*
1	172675097	rs12118303	C	T	0.19	0.17	1.12	3.02 × 10^−10^	++++++++	*FASLG*
1	206655331	rs41298997	T	C	0.19	0.18	1.13	2.37 × 10^−8^	++++++++	*IKBKE*
10	64369999	rs2944542	G	C	0.62	0.60	1.08	1.76 × 10^−8^	++++−+++	*ZNF365*
10	89824771	rs76959677	G	A	0.05	0.04	1.28	2.75 × 10^−8^	++++++?+	*PTEN, KLLN, SNORD74*
10	102038641	rs61871342	G	A	0.57	0.55	1.10	1.56 × 10^−9^	++−+++?+	*CHUK*
11	65593444	rs118086960	T	A	0.49	0.47	1.12	6.89 × 10^−9^	++++++?+	*CFL1, FIBP, FOSL1*
12	10597207	rs11053802	T	C	0.69	0.67	1.11	4.17 × 10^−9^	++++++?+	*KLRK1, KLRC4*
12	112059557	rs11065979	T	C	0.47	0.45	1.08	1.67 × 10^−8^	++−+++++	*BRAP, MAPKAPK5*
12	122668326	rs11059675	A	G	0.48	0.46	1.10	1.50 × 10^−8^	+++−++?+	*IL31*
13	99950260	rs9513593	G	A	0.19	0.18	1.12	3.60 × 10^−8^	+++−++++	*UBAC2, RN7SKP9*
14	98668778	rs142903734	AAG	A	0.81	0.79	1.12	7.15 × 10^−9^	++++++++	*RP11-61O1.1*
15	31637666	rs28624578	T	C	0.85	0.83	1.18	9.22 × 10^−10^	++++++?+	*KLF13*
17	73890363	rs55823223	A	G	0.15	0.13	1.15	1.06 × 10^−8^	++++++++	*TRIM47, TRIM65*
18	12857002	rs559406	G	T	0.47	0.45	1.10	1.19 × 10^−10^	++−+++++	*PTPN2*
19	49206417	rs492602	G	A	0.49	0.46	1.11	6.57 × 10^−13^	++++++++	*FUT2*

^*^Direction of the effect of the risk allele in the eight data sets in the order of: PsA GWAS, CASP GWAS, Kiel GWAS, Genizon GWAS, WTCCC2, Exomechip w/GWAS content, PAGE Immunochip and 23andMe GWAS, proceeding from left to right. ‘?' means the marker is not imputed well in the corresponding cohort. NRA, Non-risk allele; OR, odds ratio; RA, risk allele; RAF, risk allele frequency.
